# Comparison of Bolus and Continuous Hydration Regimens for the Prevention of Contrast‐Associated Acute Kidney Injury in the Emergency Department: A Randomized Controlled Noninferiority Trial

**DOI:** 10.1155/emmi/8497484

**Published:** 2026-07-30

**Authors:** Yunus Emre Gemici, Furkan Ay, Alper Görkem Cimen, İbrahim Sarbay, Mustafa Calik

**Affiliations:** ^1^ Department of Emergency Medicine, Istanbul Medeniyet University, Göztepe Prof. Dr. Süleyman Yalçın City Hospital, Istanbul, Türkiye, medeniyet.edu.tr; ^2^ Department of Emergency Medicine, Gaziosmanpaşa Training and Research Hospital, İstanbul, Türkiye

**Keywords:** acute kidney injury, contrast media, emergency medicine, hydration therapy, tomography

## Abstract

**Objectives:**

The optimal rate and duration of isotonic hydration therapy for preventing contrast‐associated acute kidney injury (CA‐AKI) remain poorly established. As prolonged hydration therapy is impractical due to the urgency of diagnostic imaging and the limited time available for monitoring in the emergency departments (EDs), this study evaluated whether bolus hydration regimens are noninferior to prolonged hydration protocols.

**Methods:**

This single‐center, prospective, open‐label, randomized controlled, noninferiority trial was conducted at the ED of a training and research hospital between August 10, 2024, and April 26, 2025. 257 patients undergoing contrast‐enhanced computed tomography (CT) with baseline creatinine levels above the reference range (1.2 mg/dL in men and 1.1 mg/dL in women) were included. Patients were stratified by age and sex and randomized to bolus or continuous hydration. The bolus group received 500 mL of isotonic fluid within 30 min before the procedure, followed by 500 mL/hour for 2 h postprocedure. The continuous group received 150 mL/hour for 2 h before and 8 h after the procedure. The primary outcome was the development of CA‐AKI, defined as an increase in serum creatinine of ≥ 25% or ≥ 0.5 mg/dL at 48–72 h following contrast administration. Secondary outcomes included 30‐day all‐cause mortality and the need for dialysis.

**Results:**

The two groups were well‐matched in terms of demographic, clinical, or biochemical parameters. The mean baseline creatinine was 1.4 ± 0.21 mg/dL, and the eGFR was 45.6 ± 9.8 mL/min/1.73 m^2^. CA‐AKI occurred in 1.7% of the continuous group and 4.9% of the bolus group (absolute risk difference 3.2%; 95% CI −1.3% to 7.7%). As the upper confidence interval limit was below the predefined noninferiority margin of 8%, bolus hydration was noninferior to continuous hydration for CA‐AKI prevention. No patients required dialysis. Thirty‐day all‐cause mortality was comparable (7.6% vs. 7.9%; *p* = 0.928). Significant improvements were observed in urea, creatinine, and eGFR values in both groups at 48–72 h (*p* < 0.001).

**Conclusion:**

In this single‐center noninferiority trial, a bolus hydration regimen was noninferior to a prolonged continuous regimen for preventing CA‐AKI in ED patients with moderately impaired baseline renal function. Bolus hydration may offer a practical alternative in time‐constrained ED settings; however, multicenter confirmation is warranted before routine adoption.

**Trial Registration:** ClinicalTrials.gov.identifier: NCT07286526.

**Highlights**
• This study demonstrates that bolus hydration therapy is noninferior to prolonged hydration in preventing CA‐AKI in a tertiary emergency department setting.
• Both treatment groups showed significant improvement in renal function tests.
• No significant differences were observed between the groups in terms of 30‐day all‐cause mortality or need for dialysis.

## 1. Introduction

### 1.1. Background

Acute kidney injury (AKI) is a common, preventable condition that contributes significantly to morbidity, mortality, and healthcare costs. It is characterized by a reduction in urine output and changes in renal function markers. As a global health concern, AKI is associated with both short‐ and long‐term adverse clinical outcomes [1, 2].

In developing countries, where access to timely and appropriate treatment may be limited, AKI more frequently progresses to end‐stage renal disease, thereby negatively impacting health outcomes and economic indicators. Early recognition and management of AKI during its reversible stages are particularly important in these settings, as this may reduce the progression of disease and the need for dialysis [3, 4].

CA‐AKI is a significant cause of AKI arising from the nephrotoxic effects of contrast media. Various methodologies persist concerning the diagnostic criteria for CA‐AKI. Recent literature defines CA‐AKI as *a* ≥ 25% elevation in serum creatinine levels or an absolute increase of ≥ 0.5 mg/dL within 48–72 h following exposure to contrast media [5–8].

CA‐AKI is recognized as the third most common cause of hospital‐acquired AKI and is responsible for approximately 11% of all in‐hospital AKI cases. Other common causes include reduced renal perfusion and the use of nephrotoxic medications [9]. Several studies have demonstrated that patients who develop CA‐AKI are at increased risk of in‐hospital mortality and prolonged length of stay [10].

Worldwide, the amount of imaging performed using contrast agents is increasing, and a large portion of these procedures are being carried out on outpatients with no known risk factors. With the increase in life expectancy over the years, the exposure of the population with comorbidities such as aging, chronic kidney disease (CKD), and diabetes mellitus (DM) to contrast agents has increased; consequently, the monitoring of these patient groups, who are at high risk for developing CA‐AKI, has gained importance [11].

The increasing use of contrast media in emergency departments (EDs) has led to a corresponding rise in CA‐AKI incidence, highlighting the need for effective prophylactic strategies. Most existing studies in this field have focused on patients undergoing coronary angiography and have relied on prolonged pre‐ and postprocedural hydration protocols [5, 8]. However, the urgent nature of diagnostic processes in EDs limits the feasibility of extended hydration before imaging, and the typically short ED length of stay poses a challenge for postprocedure hydration. These limitations necessitate the development of hydration protocols tailored to ED conditions.

The primary aim of this study is to compare the effectiveness of an ED‐appropriate bolus hydration regimen with a traditional long‐duration hydration protocol in the prevention of CA‐AKI under ED conditions. Additionally, the study seeks to identify risk factors for CA‐AKI in ED patients with elevated baseline serum creatinine levels undergoing contrast‐enhanced imaging, in order to inform and prioritize prophylactic treatment strategies.

## 2. Methods

### 2.1. Study Design and Settings

A single‐center, prospective, open‐label, noninferiority randomized controlled clinical trial was conducted at the ED of a tertiary hospital. The ED staff consisted of a mix of emergency medicine specialists, emergency medicine residents, and general practitioners. The ED admits all trauma patients regardless of age, as well as nontrauma patients aged 18 years and older. An average of 385,000 patients are admitted annually, and imaging with contrast media is performed on an average of 12,250 patients. The recruitment period was August 10, 2024, to March 26, 2025; the 30‐day postprocedure follow‐up of the last enrolled patient was completed on April 26, 2025.

### 2.2. Study Enrollment

Between August 10, 2024, and March 26, 2025, patients aged 18 and over who presented to the ED with creatinine levels above the reference range (1.2 mg/dL in men and 1.1 mg/dL in women) and underwent contrast‐enhanced computed tomography (CT) were deemed eligible for inclusion in the study. The 30‐day follow‐up of the last enrolled patients was completed by April 26, 2025; this date is reported as the end of the study period in the abstract. Exclusion criteria were as follows [1]: pregnancy [2], known allergy history to contrast agents [3], exposure to contrast agents within the last 72 h [4], being on dialysis due to end‐stage kidney disease [5], presenting with decompensated heart failure [6], and patients who were unable to provide informed consent. Volume status was assessed clinically by the treating emergency physician at the time of enrollment, primarily for the purpose of identifying decompensated heart failure (an exclusion criterion). Standardized formal assessments of volume status, such as inferior vena cava ultrasonography, central venous pressure measurement, or validated volume‐status scoring, were not performed; this approach reflects routine ED practice but is acknowledged as a limitation.

Eligible patients were randomly allocated in a 1:1 ratio to receive either bolus hydration or continuous hydration therapy. A permuted block randomization sequence with a fixed block size of two was generated using Microsoft Excel by one of the investigators, stratified by age group (18–44, 45–59, 60–74, and ≥ 75 years) and sex. The allocation sequence was administered through a study‐specific Google Form completed by the enrolling investigator at the time of patient inclusion. Formal allocation concealment (e.g., sequentially numbered, sealed opaque envelopes or a centralized web‐based system independent of the investigators) was not implemented; we acknowledge this as a methodological limitation. The trial was open‐label, as the difference in infusion rate and duration between the two regimens precluded blinding of patients and treating clinicians. However, the primary outcome was based on an objective laboratory measurement (serum creatinine), and laboratory personnel performing creatinine measurements were unaware of treatment allocation, which limits the potential for outcome assessment bias.

### 2.3. Interventions

After the patients were divided into two groups, one group received bolus hydration therapy, while the other group received continuous hydration therapy.

In the bolus hydration therapy group, 500 mL of 0.9% saline treatment was started half an hour before the procedure, and after the procedure, 1000 mL of 0.9% saline treatment was continued at a rate of 500 mL/h to be completed in 2 h. A total of 1500 mL 0.9% saline hydration therapy was administered over 2.5 h.

In the continuous hydration therapy group, saline treatment started 2 h before the intravenous (IV) contrast agent application at a rate of 150 mL/h, and hydration was applied for 8 more hours after the procedure at the same rate. A total of 1500 mL 0.9% saline hydration therapy was administered over 10 h.

**FIGURE 1:**
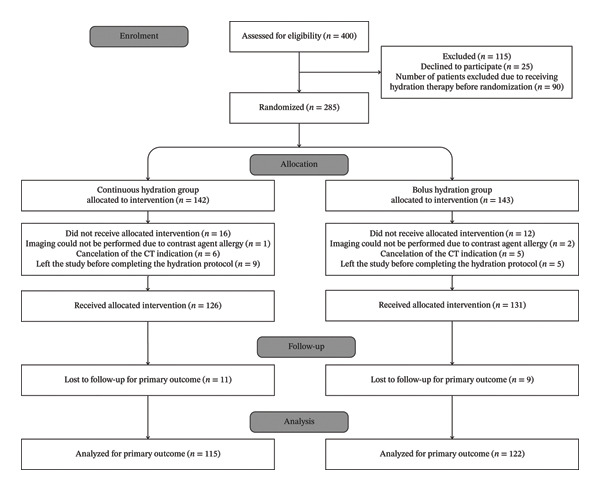
CONSORT 2010 flow diagram of patient enrollment, allocation, follow‐up, and analysis. Of 400 patients assessed for eligibility, 285 were randomized (142 to continuous hydration, 143 to bolus hydration). Twenty‐eight patients did not receive the allocated intervention, leaving 257 patients (continuous: *n* = 126; bolus: *n* = 131) who received protocolized hydration. Twenty patients were subsequently lost to follow‐up for the primary outcome assessment (continuous: *n* = 11; bolus: *n* = 9), yielding a primary analysis population of 237 patients (continuous: *n* = 115; bolus: *n* = 122).

According to the literature, individuals with an ejection fraction of less than 40% in their medical records received a half‐dose hydration protocol [12, 13]. In the bolus hydration group, 250 mL of treatment was administered half an hour before the procedure, and hydration was continued at a rate of 250 mL/h for 2 h after the procedure. In the continuous hydration group, 75 mL/h of hydration was administered for 2 h before the procedure and for 8 h after the procedure. During and after the hydration protocol, patients were monitored clinically by the treating emergency physicians for signs of fluid overload, including new‐onset dyspnea, worsening peripheral edema, and clinical features of decompensated heart failure. No additional standardized co‐interventions were administered as part of the trial protocol; all aspects of clinical care other than the assigned hydration regimen followed the treating clinicians’ routine ED practice. Among the 257 patients who received the allocated hydration regimen, full protocol adherence was 100%; patients who did not complete the assigned hydration regimen are accounted for in the CONSORT diagram (Figure 1) as “Left study before completing the hydration protocol” and were not included in the analysis.

The IV contrast agent used in the study was iohexol, which belongs to the nonionic low‐osmolar contrast agent group. 300 mgI/mL 100 mL solutions were used, and the dose was adjusted between 80 and 100 mL depending on the imaging and the patient. In the majority of patients, 100 mL of contrast was administered, with an average of 98 mL of solution given in the bolus hydration group and an average of 96 mL of solution given in the continuous hydration group.

Patients were given control forms to provide follow‐up blood samples 48–72 h later and were asked to present to the ED with the form on the specified date. To inquire about the development of dialysis needs and mortality status, patients were contacted 30 days later to gather information.

## 3. Outcomes

The primary outcome of our study was the development of CA‐AKI, defined using a conventional CA‐AKI definition commonly applied in previous contrast nephropathy trials, namely, an increase in serum creatinine value by ≥ 25% compared to baseline or an absolute increase in serum creatinine value by ≥ 0.5 mg/dL within 48–72 h after contrast administration [10].

Urine output criteria for AKI were not incorporated because reliable measurement of 6–12‐h urine output is not feasible for the majority of ED patients in our setting, where most patients undergoing contrast‐enhanced CT are subsequently discharged from the ED rather than admitted, and ED throughput and crowding preclude protocolized hourly urine output recording. This trial was deliberately designed to test a strategy applicable to routine ED conditions; the primary outcome was therefore based exclusively on the serum creatinine criterion.

The secondary outcomes of the study are the need for dialysis and all‐cause mortality within 30 days.

### 3.1. Statistical Analysis

Categorical variables are presented as frequencies and percentages. Continuous variables were assessed for normality using the Kolmogorov–Smirnov and Shapiro–Wilk tests; non‐normally distributed variables are reported as median (25th–75 th percentiles). Between‐group comparisons of categorical variables were performed using Pearson’s chi‐square or Fisher’s exact test, as appropriate. Continuous variables were compared using the Mann–Whitney *U* test for independent groups and the Wilcoxon signed‐rank test for paired groups. The incidence of contrast‐associated acute kidney injury (CA‐AKI) between hydration protocols was compared using risk differences (RDs) with 95% confidence intervals (CIs). Noninferiority of the bolus hydration protocol was assessed using a prespecified margin; the protocol was considered noninferior if the upper bound of the 95% CI was below this margin. All other statistical tests were two‐sided, and statistical significance was set at *p* < 0.05. Analyses were conducted using IBM SPSS Statistics.

*Analysis of populations and handling of missing data.* A total of 285 eligible patients were randomly allocated (continuous: *n* = 142; bolus: *n* = 143). Twenty‐eight patients did not receive the allocated intervention and were therefore not exposed to the treatment under study (continuous group: contrast agent allergy, *n* = 1; cancellation of the CT indication, *n* = 6; left the study before completing the hydration protocol, *n* = 9; bolus group: contrast agent allergy, *n* = 2; cancellation of the CT indication, *n* = 5; left the study before completing the hydration protocol, *n* = 5). The 257 patients who received the allocated hydration regimen (continuous: *n* = 126; bolus: *n* = 131) constituted the safety population and were used for the descriptive analysis of baseline characteristics. The primary outcome (CA‐AKI), which required a 48‐ to 72‐h follow‐up creatinine measurement, was assessed using a complete‐case modified intention‐to‐treat (mITT) analysis: 20 of the 257 protocol‐completing patients did not return for follow‐up creatinine sampling (continuous: *n* = 11; bolus: *n* = 9), yielding a primary analysis population of 237 patients (continuous: *n* = 115; bolus: *n* = 122). No imputation was performed for missing primary outcome data. Because the trial was open‐label and the dropouts before treatment occurred prior to exposure, a separate per‐protocol analysis would not have differed materially from the mITT analysis described above. It should be noted that, by virtue of including only patients who both received and completed the allocated hydration regimen and returned for follow‐up creatinine sampling, our mITT analysis population is operationally equivalent to a per‐protocol analysis. We therefore considered that reporting a separate, formally labeled per‐protocol analysis would not provide additional information; this approach is in line with current guidance on the analysis of noninferiority trials, which recommends that both ITT and per‐protocol analyses be reported but acknowledges that when the two populations are operationally similar, a single analysis with appropriate transparency about its composition is acceptable. The proportion of patients with missing primary outcome data among those treated was modest (20/257; 7.8%) and was balanced between groups (continuous: 11/126, 8.7%; bolus: 9/131, 6.9%), making it unlikely that informative missingness materially biased the noninferiority conclusion. The flow of participants through each stage is summarized in the CONSORT diagram (Figure 1). To assess the robustness of the primary noninferiority conclusion against alternative assumptions about missing primary‐outcome data, three additional sensitivity analyses were performed by applying extreme imputation scenarios to the 20 patients without follow‐up creatinine values: (i) a best‐case scenario for noninferiority, in which all missing patients in the continuous group were assumed to have developed CA‐AKI and all missing patients in the bolus group were assumed not to have developed CA‐AKI; (ii) a worst‐case scenario for noninferiority, with the opposite assumptions; and (iii) a conservative tipping‐point scenario in which all missing patients in both groups were assumed to have developed CA‐AKI. The RD and 95% CI were recalculated for each scenario and compared with the prespecified 8% noninferiority margin.

### 3.2. Power Calculation

The sample size calculation aims to establish the noninferiority of bolus hydration relative to continuous hydration concerning the primary outcome, CA‐AKI. The anticipated incidence of CA‐AKI patients following continuous hydration is 6.2% [6], while the noninferiority margin, derived from clinical consensus and literature, is 8% [14]. A one‐tailed hypothesis was established, with a Type I error rate (*α*) of 0.05 and a statistical power (1‐β) of 0.80. In light of clinical settings, the dropout rate was established at 15%, resulting in a total inclusion of 266 individuals, with 133 assigned to the continuous hydration group and 133 to the bolus hydration group. Given the dropout rate, the inclusion of 113 patients in the final analysis is enough for both groups [15]. The computation was performed using the complimentary website sealedenvelope.com [16].

The 8% noninferiority margin was selected on the basis of the following considerations: (i) the variability of CA‐AKI incidence reported in published prevention trials in this clinical range (typically 5%–15% in similar populations); (ii) the modest absolute increase in risk that we judged clinically acceptable in exchange for the practical advantages of a shorter, ED‐feasible regimen, including avoidance of fluid overload in patients with marginal cardiac reserve, reduced ED length of stay, and improved feasibility of obtaining contrast‐enhanced imaging in time‐sensitive presentations; and (iii) the operational reality that a long‐duration IV hydration protocol is frequently impracticable in the ED, such that the relevant clinical alternative to a shorter regimen is often no protocolized hydration at all rather than the textbook continuous regimen. Published noninferiority trials in CA‐AKI prevention have used a wide range of margins, reflecting differences in clinical context, patient population, and the perceived clinical equipoise between the regimens compared. More conservative margins (e.g., 5%) have been used in elective settings such as the trial by Liu et al., whereas more permissive margins have been used in pragmatic comparisons of shorter versus longer regimens, including the NICIR study by Sebastià et al. [14] (9%) and the pre‐TAVI CTA short‐versus‐conventional hydration trial by van Mourik et al. [17] (10%). Our 8% margin lies within this published spectrum and is consistent with our pragmatic ED‐focused research question. We acknowledge that the choice of margin influences the strength of the noninferiority claim, and we therefore interpret our findings accordingly in the Discussion.

### 3.3. Trial Registration and Prespecification of the Protocol

The study protocol—including study design, primary outcome (development of CA‐AKI), eligibility criteria, randomization scheme, hydration regimens, and statistical analysis plan—was reviewed and approved by the institutional Academic Council on February 28, 2024, and subsequently by the institutional Clinical Research Ethics Committee on August 7, 2024 (Decision No. E−66291034–202.3.02–4819). Both approvals occurred prior to enrollment of the first patient on August 10, 2024. The 8% noninferiority margin was prespecified prior to enrollment of the first patient on August 10, 2024, based on variability reported in published CA‐AKI prevention trials, and was not modified after enrollment began. Prospective registration of the trial on a public clinical trials registry was not completed before enrollment, owing to the lead investigator’s inexperience with regulatory requirements for randomized trials; the trial was subsequently registered on ClinicalTrials.gov (NCT07286526). We acknowledge this departure from current best practice in trial reporting and have addressed its implications in the Limitations section.

At the conclusion of the trial, 257 patients successfully completed the treatment regimens. Twenty patients had a deficiency in the blood results necessary for the primary outcome. In the bolus group, 122 patients were eligible for the final analysis, whereas 115 patients were eligible in the continuous group. Finally, the inclusion of dropout rates achieved the necessary patient count for both groups.

## 4. Results

### 4.1. Study Subjects

During our trial, 400 patients were assessed for eligibility over a 1‐year period, and 285 of these patients were included in the randomization process. A total of 142 patients were assigned to the continuous hydration group, whereas 143 patients were assigned to the bolus hydration group. 126 patients completed the treatment protocol with continuous hydration, whereas 131 patients did so with bolus hydration. For the primary outcome, 20 patients could not provide follow‐up blood samples at 48–72 h, resulting in 237 patients being included in the analysis (Figure 1).

The prespecified half‐dose hydration protocol for patients with documented left ventricular ejection fraction below 40% was applied to 6 patients overall (5 in the bolus hydration group and 1 in the continuous hydration group). No hydration‐related adverse events—such as new‐onset pulmonary edema, worsening heart failure, or other clinical features of fluid overload—came to the attention of the treating emergency physicians during or after the hydration protocol in either treatment group, including among patients who received the half‐dose regimen.

No statistically significant difference was found in the incidence of heart failure and DM, previously established as major predisposing factors for CA‐AKI, between the two groups (Table 1) [18].

**TABLE 1:** Comparison of demographic and clinical characteristics of patients according to treatment groups.

Characteristic	Continuous hydration (*n* = 126)	Bolus hydration (*n* = 131)	*p* value
Sex, no. (%)			
Female	59 (46.8%)	60 (45.8%)	0.86
Male	67 (53.2%)	71 (54.2%)	
Diabetes mellitus, no. (%)			
Absent	62 (49.2%)	76 (58.0%)	0.15
Present	64 (50.8%)	55 (42.0%)	
Hypertension, no. (%)			
Absent	39 (31.0%)	46 (35.1%)	0.47
Present	87 (69.0%)	85 (64.9%)	
Heart failure, no. (%)			
Absent	103 (81.7%)	112 (85.5%)	0.41
Present	23 (18.3%)	19 (14.5%)	
Coronary artery disease, no. (%)			
Absent	93 (73.8%)	89 (67.9%)	0.30
Present	33 (26.2%)	42 (32.1%)	
Stroke history, no. (%)			
Absent	115 (91.3%)	119 (90.8%)	0.90
Present	11 (8.7%)	12 (9.2%)	
Patient outcome, no. (%)			
Discharge	79 (62.7%)	89 (67.9%)	0.38
Ward admission	40 (31.7%)	32 (24.4%)	
ICU admission	7 (5.6%)	10 (7.6%)	
30‐day all‐cause mortality, no. (%)			
No	116 (92.1%)	121 (92.4%)	0.92
Yes	10 (7.9%)	10 (7.6%)	
CA‐AKI development, no. (%)			
No	113 (98.3%)	116 (95.1%)	0.28^∗^
Yes	2 (1.7%)	6 (4.9%)	

*Note:* Data are presented as *n* (%). Statistical significance was defined as *p* < 0.05. Pearson’s chi‐square test was used. Demographic and clinical characteristics are presented for the 257 patients who completed the assigned hydration protocol (continuous: *n* = 126; bolus: *n* = 131). The CA‐AKI primary outcome was assessed in the 237 patients with available 48‐ to 72‐h follow‐up creatinine values (continuous: *n* = 115; bolus: *n* = 122); for the CA‐AKI row, percentages are calculated using these denominators.

Abbreviations: CA‐AKI, contrast‐associated acute kidney injury; ICU, intensive care unit.

^∗^Fisher’s exact test was applied where indicated.

No statistically significant differences were observed between the two groups in key biochemical and clinical parameters relevant to CA‐AKI predisposition, including age, blood pressure, baseline urea/creatinine/eGFR, hemoglobin, and glucose levels (Table 2) [18].

**TABLE 2:** Comparison of biochemical characteristics of patients according to treatment groups.

Variable	Continuous hydration (*n* = 126) median (IQR)	Bolus hydration (*n* = 131) median (IQR)	*p* value
Age (years)	73 (61–79)	72 (60–80)	0.85
Height (cm)	165 (160–173)	166 (160–175)	0.50
Weight (kg)	80 (70–88)	80 (65–90)	0.40
BMI (kg/m^2^)	29 (24.8–32.2)	27.7 (24.2–31.3)	0.14
MAP (mmHg)	94 (85–103)	93 (83–102)	0.44
SpO_2_ (%)	96 (94–98)	97 (95–98)	0.73
Creatinine (mg/dL)			
Baseline (0 h)	1.32 (1.24–1.48)	1.36 (1.25–1.52)	0.27
72 h	1.16 (1.01–1.35)	1.21 (1.04–1.43)	0.41
% Change	−10.3 (−23.8 to −2.9)	−13.9 (−24.3 to 0.8)	0.93
Absolute difference	−0.14 (−0.32 to −0.05)	−0.18 (−0.34 to 0.01)	0.94
Urea (mg/dL)			
Baseline (0 h)	58.7 (44.5–72.7)	58.5 (45.4–77.3)	0.66
72 h	46.9 (34.6–64.0)	45.9 (33.0–63.1)	0.60
% Change	−18.1 (−32.5 to 2.3)	−18.5 (−33.0 to 2.7)	0.84
Absolute difference	−10.8 (−21.4 to 0.9)	−9.3 (−21.4 to 1.7)	0.72
eGFR (mL/min/1.73 m^2^)			
Baseline (0 h)	45.6 (39.8–53.0)	45.3 (37.5–51.7)	0.41
72 h	54.6 (44.4–68.5)	53.9 (42.6–66.9)	0.47
% Change	13.8 (3.5–36.8)	18.8 (0–37.9)	0.91
Absolute difference	6.2 (1.3–17.7)	9.7 (0–17.2)	0.83
Hematologic parameters			
Hemoglobin (g/L)	132 (115–142)	125 (110–142)	0.25
Hematocrit (%)	39.9 (34.3–43.1)	39.0 (34.5–42.7)	0.41
WBC (× 10^9^/L)	10.8 (8.0–13.7)	9.4 (7.4–13.6)	0.26
Platelets (× 10^9^/L)	227 (190–275)	234 (188–298)	0.41
Electrolytes			
Sodium (mmol/L)	139 (136–141)	138 (136–141)	0.75
Chloride (mmol/L)	103.5 (101–106)	105 (102–107)	0.10
Potassium (mmol/L)	4.41 (4.01–4.72)	4.40 (4.03–4.74)	0.97
Other			
Glucose (mg/dL)	139.5 (115–188)	136 (109–180)	0.71
CRP (mg/L)	38.5 (10–102)	25 (6–92)	0.13
Mehran risk score	7 (5–10)	8 (5–11)	0.34

*Note:* SpO_2_: Peripheral oxygen saturation. WBC: white blood cell count. Data are presented as median (IQR). Statistical significance was defined as *p* < 0.05. Mann–Whitney *U* test was used.

Abbreviations: BMI, body mass index; BP, blood pressure; CRP, C‐reactive protein; eGFR, estimated glomerular filtration rate; MAP, mean arterial pressure.

As presented in Table 3, our analysis revealed no statistically significant correlation between sex, the occurrence of comorbidities, patient outcome, 30‐day all‐cause mortality, and CA‐AKI.

**TABLE 3:** Distribution of patient characteristics according to the development of CA‐AKI.

Characteristic	No CA‐AKI	CA‐AKI	*p* value
Sex, no. (%)			
Female	104 (97.2%)	3 (2.8%)	0.73
Male	125 (96.2%)	5 (3.8%)	
Treatment group, no. (%)			
Continuous hydration	113 (98.3%)	2 (1.7%)	0.28
Bolus hydration	116 (95.1%)	6 (4.9%)	
Presence of diabetes mellitus, no. (%)			
No	121 (96.0%)	5 (4.0%)	0.72
Yes	108 (97.3%)	3 (2.7%)	
Presence of hypertension, no. (%)			
No	78 (96.3%)	3 (3.7%)	1
Yes	151 (96.8%)	5 (3.2%)	
Presence of heart failure, no. (%)			
No	192 (96.5%)	7 (3.5%)	1
Yes	37 (97.4%)	1 (2.6%)	
Presence of coronary artery disease, no. (%)			
No	167 (97.7%)	4 (2.3%)	0.22
Yes	62 (93.9%)	4 (6.1%)	
History of stroke, no. (%)			
No	207 (96.3%)	8 (3.7%)	0.45
Yes	22 (100.0%)	0 (0.0%)	
Patient outcome, no. (%)			
Discharged	148 (96.7%)	5 (3.3%)	1
Hospitalization	81 (96.4%)	3 (3.6%)	
30‐Day all‐cause mortality, no. (%)			
No	214 (97.3%)	6 (2.7%)	0.10
Yes	15 (88.2%)	2 (11.8%)	

*Note:* Statistical significance was set at *p* < 0.05. Analyses were performed using Fisher’s exact test.

Abbreviation: CA‐AKI: contrast‐associated acute kidney injury.

CA‐AKI occurred in two of 115 patients (1.7%) in the continuous hydration group and in six of 122 patients (4.6%) in the bolus hydration group, corresponding to an RD of 3.2%. The 95% CI for the RD was −1.3% to 7.7%. As the upper bound was below the prespecified noninferiority margin of 8%, the bolus hydration protocol was considered noninferior to the conventional continuous regimen for preventing CA‐AKI in this ED population (Table 4).

**TABLE 4:** Incidence of CA‐AKI by hydration protocol.

Treatment group	CA‐AKI no, *n* (%)	CA‐AKI Yes, *n* (%)	Total, *n*	Risk difference (bolus − continuous)	95% CI
Continuous hydration	113 (98.3%)	2 (1.7%)	115	—	—
Bolus hydration	116 (95.1%)	6 (4.9%)	122	3.2%	−1.3%–7.7%

Abbreviation: CA‐AKI: contrast‐associated acute kidney injury.

Prespecified sensitivity analyses for the 20 patients with missing 48‐ to 72‐h follow‐up creatinine values produced the following results. Under the best‐case scenario for noninferiority (all 11 missing patients in the continuous group assumed to have developed CA‐AKI and all 9 missing patients in the bolus group assumed not to have developed CA‐AKI), CA‐AKI rates were 13/126 (10.3%) in the continuous group and 6/131 (4.6%) in the bolus group; the RD was −5.7% (95% CI: −12.1%–0.7%), and noninferiority was maintained. Under the conservative tipping‐point scenario in which all 20 missing patients in both groups were assumed to have developed CA‐AKI, CA‐AKI rates were 13/126 (10.3%) and 15/131 (11.4%), respectively; the RD was 1.1% (95% CI: −6.5%–8.7%), and the upper bound of the CI modestly exceeded the 8% margin. Under the worst‐case scenario for noninferiority (all 9 missing patients in the bolus group assumed to have developed CA‐AKI and all 11 missing patients in the continuous group assumed not to have developed CA‐AKI), CA‐AKI rates were 2/126 (1.6%) and 15/131 (11.4%); the RD was 9.9% (95% CI: 4.0%–15.7%), and noninferiority was not maintained. Taken together, these sensitivity analyses indicate that the noninferiority conclusion is preserved under the best‐case and primary mITT analyses but is sensitive to extreme assumptions about the missing data, particularly the worst‐case scenario. The implications of these findings are addressed in the Discussion and Limitations sections.

In Table 5, statistically significant decreases in creatinine and urea, and a statistically significant increase in eGFR values were observed in both therapy groups at 48–72 h post‐treatment.

**TABLE 5:** Comparison of baseline and 48‐ to 72‐hour creatinine, urea, and eGFR values by treatment groups.

Parameter		0‐h median (25P–75P)	48‐ to 72‐h median (25P–75P)	*p* value
Continuous hydration	Creatinine (mg/dL)	1.32 (1.24–1.48)	1.16 (1.01–1.35)	**< 0.001**
	Urea (mg/dL)	58.72 (44.48–72.66)	46.86 (34.56–64.00)	**< 0.001**
	eGFR (mL/min/1.73 m^2^)	45.6 (39.8–53.0)	54.6 (44.4–68.5)	**< 0.001**

Bolus hydration	Creatinine (mg/dL)	1.36 (1.25–1.52)	1.21 (1.04–1.43)	**< 0.001**
	Urea (mg/dL)	58.49 (45.37–77.34)	45.93 (32.98–63.10)	**< 0.001**
	eGFR (mL/min/1.73 m^2^)	45.3 (37.5–51.7)	53.9 (42.6–66.9)	**< 0.001**

*Note:* Statistical significance was defined as *p* < 0.05. Analyses were performed using the Wilcoxon signed‐rank test. Bold values indicate statistical significance (*p* < 0.05).

Abbreviation: eGFR: estimated glomerular filtration rate.

**FIGURE 2:**
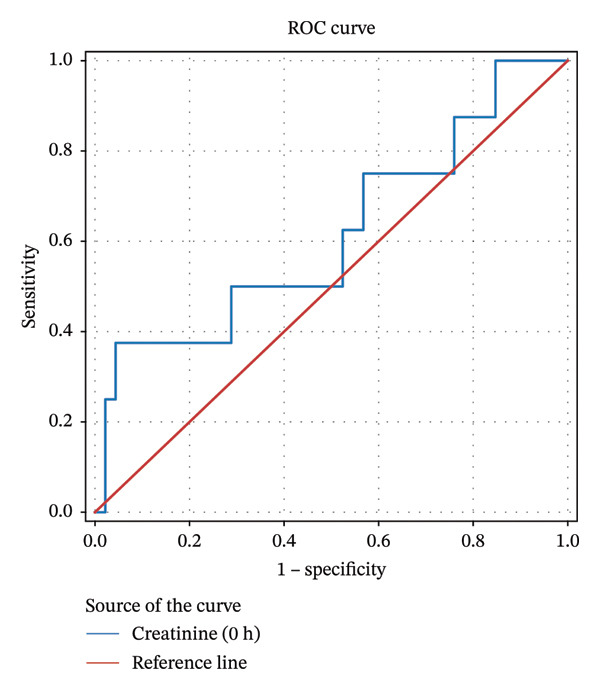
ROC curve analysis for baseline creatinine in predicting CA‐AKI.

ROC analysis was performed to evaluate the predictive performance of baseline creatinine for CA‐AKI diagnosis. The area under the curve (AUC) was 0.624, with a cutoff value of 1.225 mg/dL yielding 87.5% sensitivity and 3.6% specificity (Table 6 and Figure 2).

**TABLE 6:** ROC curve analysis for baseline creatinine in predicting CA‐AKI.

Variable	AUC	Cutoff value (mg/dL)	Sensitivity (%)	Specificity (%)
Creatinine (0 h)	0.624	1.225	87.5	3.6

## 5. Discussion

To our knowledge, our study is the first ED‐based randomized clinical trial to demonstrate that an ED‐appropriate bolus hydration regimen for preventing CA‐AKI is noninferior to long‐term hydration therapies. In our study, two different hydration regimens were tested: a 2.5‐h bolus group and a 10‐h continuous group. While the continuous hydration group provided a regimen closer to the guideline recommendations, the duration was significantly shortened in the bolus group. Additionally, the infusion rates for hydration therapy in both groups are high, particularly in the bolus treatment group, according to guideline recommendations [19, 20].

The non‐inferiority finding should, however, be interpreted with appropriate caution. The absolute RD for CA‐AKI was 3.2% in favor of the continuous regimen, with a 95% CI of −1.3% to 7.7%. Although the upper bound of this interval lay below the prespecified 8% noninferiority margin and therefore satisfied the formal criterion for noninferiority, the upper bound was close to the prespecified margin, and the relatively small number of CA‐AKI events in this trial limits precision. Results that fall close to the prespecified margin are sensitive to small variations in event rates, missing data assumptions, and the choice of analysis population, and they do not provide the same strength of evidence as a CI that is well within the noninferiority zone. The prespecified sensitivity analyses for the missing primary‐outcome data, presented in the Results, are directly consistent with this cautious interpretation. While the best‐case scenario and the primary mITT analysis both supported noninferiority, the conservative tipping‐point scenario (assuming CA‐AKI in all 20 missing patients) yielded an upper confidence‐interval bound that modestly exceeded the 8% margin, and the worst‐case scenario for noninferiority did not maintain it. Although the worst‐case scenario is clinically implausible (it assumes that all missing patients in the bolus group developed CA‐AKI while none of the missing patients in the continuous group did), the dependence of the conclusion on the imputation assumptions reinforces that this trial should be regarded as supportive but not definitive evidence for the non‐inferiority of bolus hydration. Accordingly, the conclusion of noninferiority should be regarded as suggestive rather than definitive, and replication in larger, multicenter trials with adequately powered subgroup analyses is needed before this finding can be considered robust enough to inform routine practice changes.

As a result of our study, CA‐AKI developed in 6 patients in the bolus hydration group (4.9%) and in 2 patients in the continuous hydration group (1.7%), and no statistically significant difference was found between the two groups (*p* = 0.282). No patients developed a need for dialysis within the 30‐day period. Thirty‐day all‐cause mortality occurred in 10 patients in each group (7.9% in the continuous group and 7.6% in the bolus group), with no statistically significant difference between groups (*p* = 0.928). In the comparison of creatinine, urea, and eGFR values at 0 and 48–72 h for the patients, a statistically significant decrease was found for all three parameters (*p* < 0.001). In the bolus and continuous treatment groups, these parameters were evaluated separately at 0 and 48–72 h, and a statistically significant decrease was found in three parameters in both treatment groups (*p* < 0.001). These statistically significant alterations underscore the necessity of providing hydration therapy to at‐risk patients in ED settings. A considerable number of patients arriving at the ED have underlying AKI, which exacerbates contrast nephropathy [21]. These decreases between 0 and 72 h in kidney function tests indicate that patients presented to the ED with high urea/creatinine values compared to their baseline values and benefited from treatment.

The observed improvement in serum creatinine and eGFR at 48–72 h in both treatment groups should, however, be interpreted with caution, and several alternative or contributory explanations deserve consideration. First, many ED patients present with a degree of pre‐renal azotemia driven by acute illness, dehydration, reduced oral intake, or vomiting; baseline creatinine measured at presentation may therefore overestimate the patient’s true steady‐state value, and any subsequent rehydration—whether protocolized study hydration or routine clinical fluid management—will lead to apparent “improvement” through resolution of pre‐renal physiology rather than a true treatment effect on contrast‐induced injury. This is a form of regression to the mean and is well described in studies that enroll patients on the basis of an elevated index laboratory value. Second, patients admitted to the ward or intensive care unit (ICU) (a minority of our cohort: 31.7% in the continuous group and 32.0% in the bolus group, including ICU admissions) may have received additional IV fluids, nutritional support, or modifications of nephrotoxic medication during the 48‐ to 72‐h follow‐up window, which could independently contribute to improvement in renal indices; we did not capture detailed post‐ED in‐hospital management. Third, however, several features of our cohort argue against attributing the observed improvement entirely to nonhydration confounders: the majority of our patients (approximately 65%) were discharged from the ED and therefore did not receive systematic post‐ED inpatient hydration, the disposition pattern (discharge, ward admission, ICU admission) was balanced between the two treatment groups (*p* = 0.38), and the magnitude of improvement was similar in the bolus and continuous groups despite very different total fluid volumes administered during the protocolized period. Taken together, the changes in renal indices over 48–72 h are most consistent with a combined contribution of resolution of pre‐renal physiology and protocolized hydration, with the relative contribution of each not separable in this study design. We have flagged the unmeasured contribution of post‐ED in‐hospital management as a limitation.

Most studies in the literature have tested different hydration protocols in patients undergoing coronary angiography. In a retrospective study by Chen et al. involving patients undergoing elective coronary angiography, pre‐ and postprocedure long‐term hydration regimens (24 h) were compared, revealing rates of CA‐AKI at 3.54% and 4.8%, respectively. The average GFR for the group of patients is 105 mL/min/1.73 m^2^, which is very good based on our research [5]. Liu et al.’s multicenter randomized controlled trial indicates that the baseline renal parameters of the population are comparable to those in our study (mean GFR 45 mL/min/1.73 m^2^). This study examined 5‐ vs 24‐h hydration regimes, revealing CA‐AKI rates of 6.2%–8.4% in both groups [6]. Arslan et al. reported a CA‐AKI incidence of 7.1% among patients who underwent more than 27 h of hydration [8]. The low rates of CA‐AKI in our investigation indicate that short‐term hydration protocols may be comparable to those in the literature. It is important to note that individuals having coronary angiography are susceptible to suffering AKI, and comparisons between studies should be conducted accordingly.

In our study, 5 of 8 patients (62.5%) diagnosed with CA‐AKI were discharged following assessment in the ED. This elevated rate highlights the necessity of outpatient follow‐up for patients with impaired kidney function discharged from the ED following contrast‐enhanced imaging. The favorable clinical status of this discharged patient group diminishes their probability of requiring hydration therapy.

According to ROC analysis, a threshold value of 1.225 mg/dL for creatinine yielded 87.5% sensitivity and 3.6% specificity. Using a threshold value with elevated sensitivity is advisable, given that the hydration treatment applied is cost‐effective, readily available, and simple to implement; the derived threshold value identifies the group of patients to be assessed for the risk of CA‐AKI. The limited specificity is not a significant issue for the same reasons. Based on the results of this analysis, we recommend prophylactic bolus hydration therapy for patients above the threshold value. Several important caveats apply to this ROC analysis. The AUC of 0.624 indicates only modest overall discriminative performance, and the very low specificity (3.6%) at the chosen high‐sensitivity threshold means that the identified cutoff cannot be used to rule in CA‐AKI risk on its own. The analysis is also limited by the small total number of CA‐AKI events (*n* = 8), which yields wide CIs around the AUC and threshold estimates. We therefore present this analysis as exploratory and hypothesis‐generating, intended to support a low‐threshold, broad‐application screening strategy in which prophylactic hydration is offered to all patients with serum creatinine above the upper limit of the reference range, rather than as a validated clinical decision rule. A validated risk‐stratification tool would require external derivation and validation in a substantially larger, multicenter cohort with adequate event numbers, ideally incorporating additional clinical variables (age, comorbidities, baseline eGFR, contrast volume) rather than baseline creatinine alone. In this context, recent work in higher risk contrast‐exposed cohorts—particularly patients undergoing percutaneous coronary intervention for acute coronary syndromes—has examined biochemical and angiographic predictors of CA‐AKI, including admission serum magnesium levels [22] and the combination of SYNTAX and Mehran scores with inflammatory markers [23]. Although these populations differ substantially from the ED contrast‐enhanced CT population studied here in terms of contrast dose, hemodynamic stress, and baseline cardiovascular risk profile and therefore cannot be directly extrapolated to our setting, they illustrate that CA‐AKI risk is shaped by a combination of clinical, biochemical, and procedural factors, and they support the broader argument that risk stratification based on a single renal parameter is unlikely to be sufficient.

Determining the noninferiority margin presented a methodological challenge due to the absence of standardized prophylactic hydration protocols and the substantial heterogeneity in CA‐AKI incidence reported across previous studies [9]. To ensure that the selected margin remained both statistically defensible and clinically meaningful, we adopted an 8% absolute noninferiority margin, which corresponds to the upper range of variability reported in comparable CA‐AKI prevention trials [12, 14]. This threshold was considered acceptable given the expected event rates in this population and the disease’s self‐limiting characteristics [24] was chosen to avoid masking any clinically relevant differences in hard renal outcomes [25, 26]. The incidence of CA‐AKI was less than anticipated in the power analysis. The outcome may be due to either the strong efficacy of both therapies or the population’s lower‐than‐anticipated risk level.

A direct consequence of this lower‐than‐anticipated event rate is that the relatively small number of CA‐AKI events in this trial restricts the inferences that can be drawn from subgroup analyses. We performed exploratory subgroup analyses by baseline eGFR category and by selected comorbidities; however, the trial was not powered to detect treatment‐by‐subgroup interactions, and the number of events within strata is too small to support firm conclusions about which patient subgroups are most likely to benefit from, or be harmed by, a shorter bolus regimen. These subgroup findings should therefore be regarded as hypothesis‐generating rather than confirmatory, and any clinical decisions about applying bolus hydration to specific patient subgroups (for example, patients with more advanced CKD) should await dedicated, adequately powered studies.

### 5.1. Limitations

Our study was conducted in the ED of a single training and research hospital. There may be significant differences between the conditions under which the study was conducted and those in other centers. Even though patients were ensured to receive standard hydration regimens, patients, especially those admitted to the ward or ICU, may have received additional treatments (particularly hydration therapy) during the 48‐ to 72‐h follow‐up period. Patients could not be followed up after their discharge from the ED regarding the treatments they received.

In our study, treatment regimens were administered in mL/hr. In the literature, hydration treatments are generally administered in mL/kg/hour. Although this preference was made to determine the standard hydration regimen suitable for the ED settings, it creates a limitation in terms of treatment standardization.

Blinding could not be applied because practitioners and patients could not distinguish the difference in the speed and duration of intergroup hydration treatments.

Formal allocation concealment was not implemented; allocation was administered through a study‐specific Google Form completed by the enrolling investigator. Although the open‐label design and the objective laboratory‐based primary outcome (blinded creatinine measurement) limit the potential for selection or detection bias, this departure from optimal concealment practice is a methodological limitation.

Although the study protocol—including the primary outcome, eligibility criteria, and randomization scheme—was institutionally approved prior to enrollment, the trial was not prospectively registered on a public clinical trials registry; registration on ClinicalTrials.gov (NCT07286526) was completed retrospectively. We acknowledge that retrospective registration is a deviation from current best practice in clinical trial reporting and may limit external transparency, even when the protocol itself was prespecified.

The primary outcome was analyzed using a complete‐case modified intention‐to‐treat (mITT) approach, as 20 of the 257 protocol‐completing patients did not return for the 48‐ to 72‐h follow‐up creatinine measurement. No imputation was performed for missing primary outcome data. Although the proportion of missing primary outcome data was modest (7.8%) and balanced between groups, the use of complete‐case analysis (rather than ITT with imputation) is a recognized limitation in noninferiority trials, as it can yield slightly less conservative results.

The total number of CA‐AKI events observed in this trial was lower than anticipated in the power analysis. The relatively small number of primary outcome events limits the statistical precision of both the overall noninferiority estimate and any subgroup analyses (for example, by baseline eGFR category, age, or comorbidity status). Subgroup findings should therefore be considered exploratory and hypothesis‐generating; the trial was not powered to detect treatment‐by‐subgroup interactions, and confident conclusions about differential treatment effects in particular patient subgroups would require larger trials with adequate event numbers within each stratum.

Detailed information on the use of medications that may influence renal hemodynamics or contrast‐associated kidney injury risk—such as angiotensin‐converting enzyme inhibitors (ACEIs), angiotensin II receptor blockers (ARBs), diuretics, sodium‐glucose cotransporter 2 (SGLT2) inhibitors, nonsteroidal anti‐inflammatory drugs (NSAIDs), and other potentially nephrotoxic agents—was not systematically collected. Similarly, peri‐procedural decisions to continue, hold, or modify these medications were not captured. Although randomization should, on average, balance the prevalence of such medication exposures between the two groups, the absence of these data prevents adjusted analyses and limits inference about whether specific medication exposures modify the relative effect of bolus versus continuous hydration. Future studies should systematically record concomitant nephroactive medications and any peri‐procedural medication management.

Volume status was assessed clinically by the treating emergency physician, primarily to identify and exclude patients with decompensated heart failure. Standardized formal assessments of volume status—such as inferior vena cava ultrasonography, central venous pressure measurement, or validated clinical volume‐status scoring—were not performed. As a result, we could not characterize subtler differences in baseline volume status between the two groups, and we cannot exclude the possibility that small imbalances in baseline hydration influenced the magnitude of the change in serum creatinine observed at 48–72 h.

The generalizability of our findings is limited in several respects. First, the trial enrolled patients with mildly to moderately impaired baseline renal function, and patients with severe CKD, those on dialysis, and those with decompensated heart failure were excluded by design. Our results therefore should not be extrapolated to patients with advanced CKD (e.g., eGFR < 30 mL/min/1.73 m^2^), to dialysis‐dependent patients, or to patients with active acute decompensated heart failure, in whom the balance between the benefits of hydration and the risks of fluid overload is fundamentally different. Second, the trial was conducted at a single high‐volume tertiary ED in one country, and ED case‐mix, contrast volumes, available imaging protocols, and post‐ED handover practices may differ in other settings. Third, although our pragmatic ED‐focused design enhances applicability to similar ED settings, it may limit comparability with elective contrast administration trials performed in radiology suites or cardiac catheterization laboratories. Multicenter confirmation, including dedicated subgroup recruitment of patients with more severe baseline renal impairment, will be necessary before any change in routine clinical practice can be recommended.

Hydration‐related adverse events were monitored clinically by the treating emergency physicians, and no events came to clinical attention during the trial. However, systematic prospective ascertainment of adverse events using a structured case‐report form was not performed; therefore, while we can report that no clinically apparent fluid‐overload events were observed, the absence of structured ascertainment limits our ability to report adverse‐event rates with full confidence.

Baseline proteinuria data were not systematically collected, as routine urinalysis was not part of the standardized precontrast assessment in our ED protocol. Because proteinuria is a well‐established independent risk factor for CA‐AKI, particularly in patients with hypertension, DM, or heart failure, the absence of these data prevents adjustment for proteinuria status across treatment groups. Although randomization would be expected to balance unmeasured prognostic factors on average, this remains a limitation that should be addressed in future studies, which should systematically record baseline proteinuria as part of the screening assessment.

## 6. Conclusion

In summary, hydration therapy is a straightforward, cost‐effective, and readily available intervention for use in the ED. In this single‐center noninferiority trial, a bolus hydration regimen showed comparable performance to a longer continuous regimen for preventing CA‐AKI among ED patients with moderately impaired baseline renal function. While these findings support the practical feasibility of bolus hydration in time‐constrained ED settings, broader recommendations should await confirmation in multicenter trials with larger samples and a wider range of baseline renal function. Until such evidence becomes available, individualized clinical judgment should guide the choice of hydration protocol in this patient population.

The efficacy of suitable hydration protocols in the ED should be validated through multicenter randomized controlled trials to develop a standard treatment algorithm. Furthermore, bolus hydration protocols must be evaluated against standard practices in emergency clinics to ascertain the actual efficacy of these interventions.

**Nomenclature**
AKI: Acute kidney injury
AUC: Area under the curve
BMI: Body mass index
BP: Blood pressure
CA‐AKI: Contrast‐associated acute kidney injury
CI: Confidence interval
CKD: Chronic kidney disease
CONSORT: Consolidated Standards of Reporting Trials
CRP: C‐reactive protein
CT: Computed tomography
DM: Diabetes mellitus
ED: Emergency department
eGFR: Estimated glomerular filtration rate
ICU: Intensive care unit
IQR: Interquartile range
IV: Intravenous
KDIGO: Kidney Disease: Improving Global Outcomes
RD: Risk difference
ROC: Receiver operating characteristic
SpO_2_
: Peripheral oxygen saturation
WBC: White blood cell count

## Author Contributions

Conceptualization: Yunus Emre Gemici and İbrahim Sarbay.

Data curation: Yunus Emre Gemici, Furkan Ay, and Alper Görkem Cimen.

Formal analysis: Yunus Emre Gemici.

Investigation: Yunus Emre Gemici and İbrahim Sarbay.

Methodology: Yunus Emre Gemici, İbrahim Sarbay, and Mustafa Calik.

Supervision: Mustafa Calik.

Validation: Yunus Emre Gemici and İbrahim Sarbay.

Visualization: Yunus Emre Gemici and Alper Görkem Cimen.

Writing–original draft: Yunus Emre Gemici and Furkan Ay.

Writing–review and editing: İbrahim Sarbay and Mustafa Calik.

## Funding

This research received no external funding. Minor incidental costs, such as stationery expenses, were covered by the researchers themselves.

## Ethics Statement

The study was approved by the Clinical Research Ethics Committee of T.C. Istanbul Medipol University (Decision No. E−66291034–202.3.02–4819, Date: 07/08/2024) and was conducted in accordance with the principles of the Helsinki Declaration. This study has been reported in accordance with the CONSORT statement. Informed written consent was obtained from all patients participating in the study.

## Conflicts of Interest

The authors declare no conflicts of interest.

## Data Availability

The datasets analyzed during the current study are available in the ClinicalTrials.gov repository. Additionally, the datasets are available from the corresponding author upon reasonable request.
